# Supplementary material for: Transcriptomic Landscape and Functional Characterization of Human Induced Pluripotent Stem Cell-Derived Limbal Epithelial Progenitor Cells
Source: Cells. 2022 Nov 24;11(23):3752. doi: 10.3390/cells11233752 (PMC9737332; doi:10.3390/cells11233752)
Supplement: Supplementary file 1 [file cells-11-03752-s001.zip › cells-1985946-supplementary.pdf]

Supplemental Information

Transcriptomic Landscape and Functional Characterization of Human Induced Pluripotent Stem Cell-derived Limbal Epithelial Progenitor cells

Naresh Polisetti[1*], Julian Rapp[1], Paula Liang[1], Viviane Dettmer-Monaco[2], Felicitas Bucher[1], Jan Pruszak[3,#4], Ursula Schlötzer-Schrehardt[5], Toni Cathomen[2,3], Günther Schlunck[1], Thomas Reinhard[1]

[1]Eye Center, Medical Center - Faculty of Medicine, University of Freiburg, Killianstrasse 5, 79106, Freiburg, Germany

[2]Institute for Transfusion Medicine and Gene Therapy, Medical Center – University of Freiburg, Freiburg, Germany.

[3]Freiburg iPS Core, Center for Chronic Immunodeficiency, Medical Center – University of Freiburg, Freiburg, Germany.

[#4]current address: Institute of Anatomy and Cell Biology, Paracelsus Medical University, Salzburg, Austria

[5]Department of Ophthalmology, University Hospital Erlangen, Friedrich-Alexander-University of Erlangen-Nürnberg, Schwabachanlage 6, D-91054 Erlangen, Germany

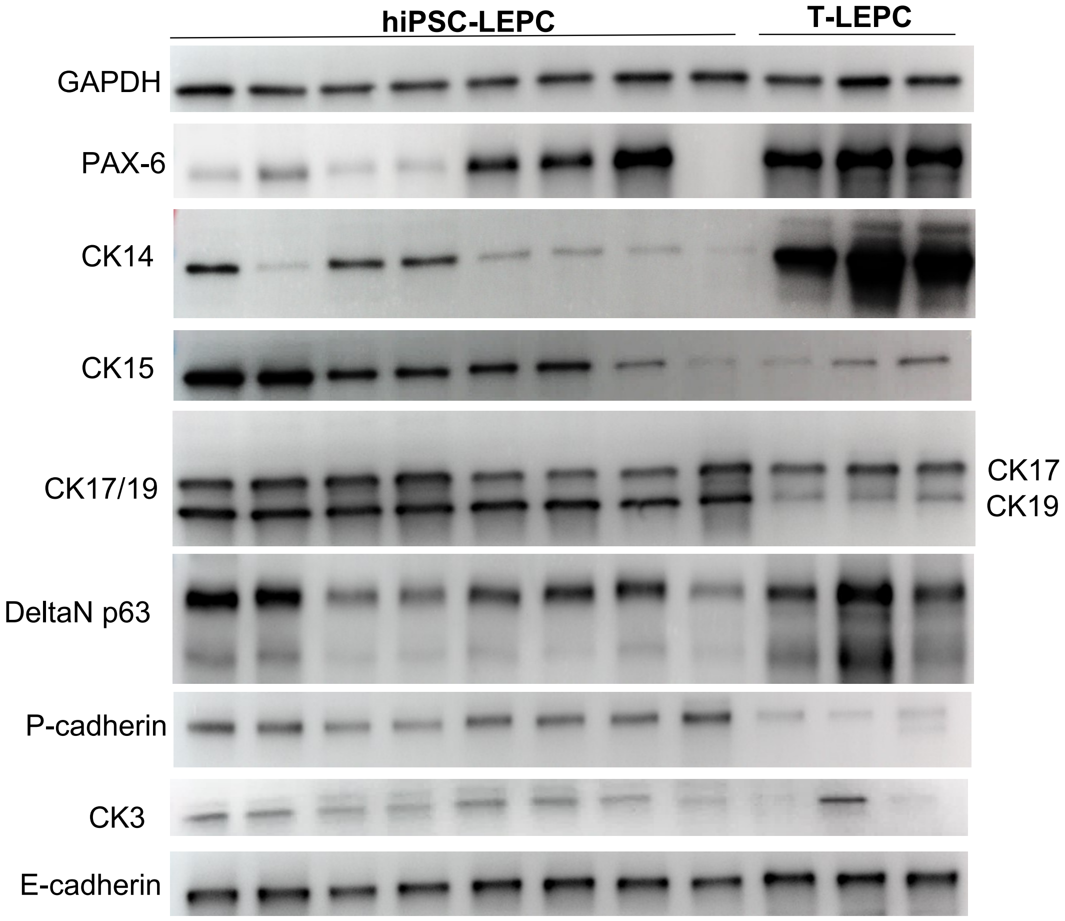

Figure S1: Western blot analysis of hiPSC-LEPC (eight individual experiments and T-LEPC (three individual experiments) showing the expression of ocular developmental marker (PAX6, 7/8 samples), limbal epithelial progenitor markers (CK14, CK15, CK17/19, DeltaN p63 (95 & 75 kDa), P-cadherin) and corneal epithelial differentiation markers (CK3 and E-cadherin). Reprobing with an anti-GAPDH antibody served as a control

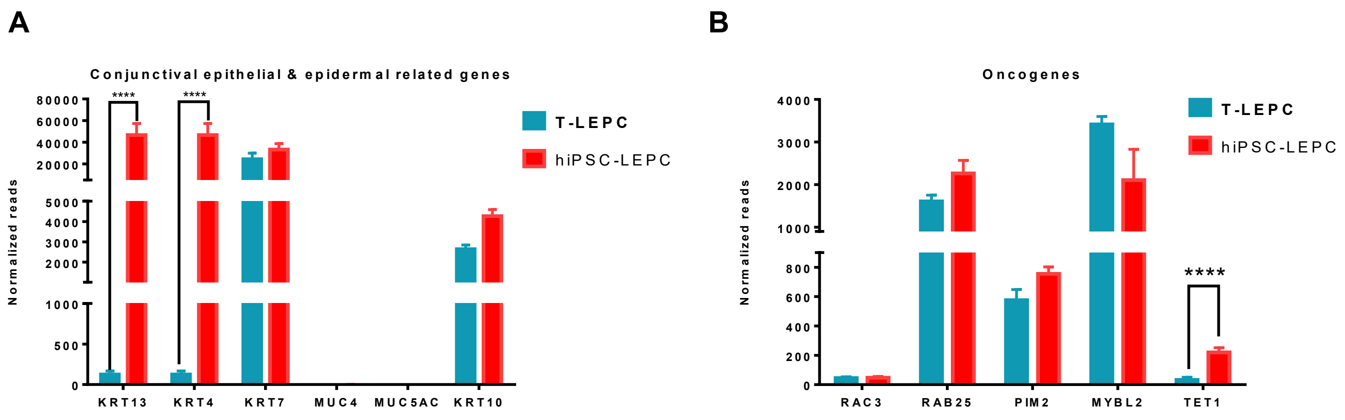

Figure S2: A) RNA sequencing analysis of hiPSC-LEPC and T-LEPC shows the expression of conjunctival and epidermal related genes. Genes considered differentially expressed log2 fold change >2.0, p<0.05. Data are expressed as mean ± standard error of the mean. ****p < 0.0001 **B)** The graph showing the expression of oncogenes in hiPSC-LEPC and T-LEPC. Genes considered differentially expressed log2 fold change >2.0, p<0.05. Data are expressed as mean ± standard error of the mean. ****p < 0.0001

A

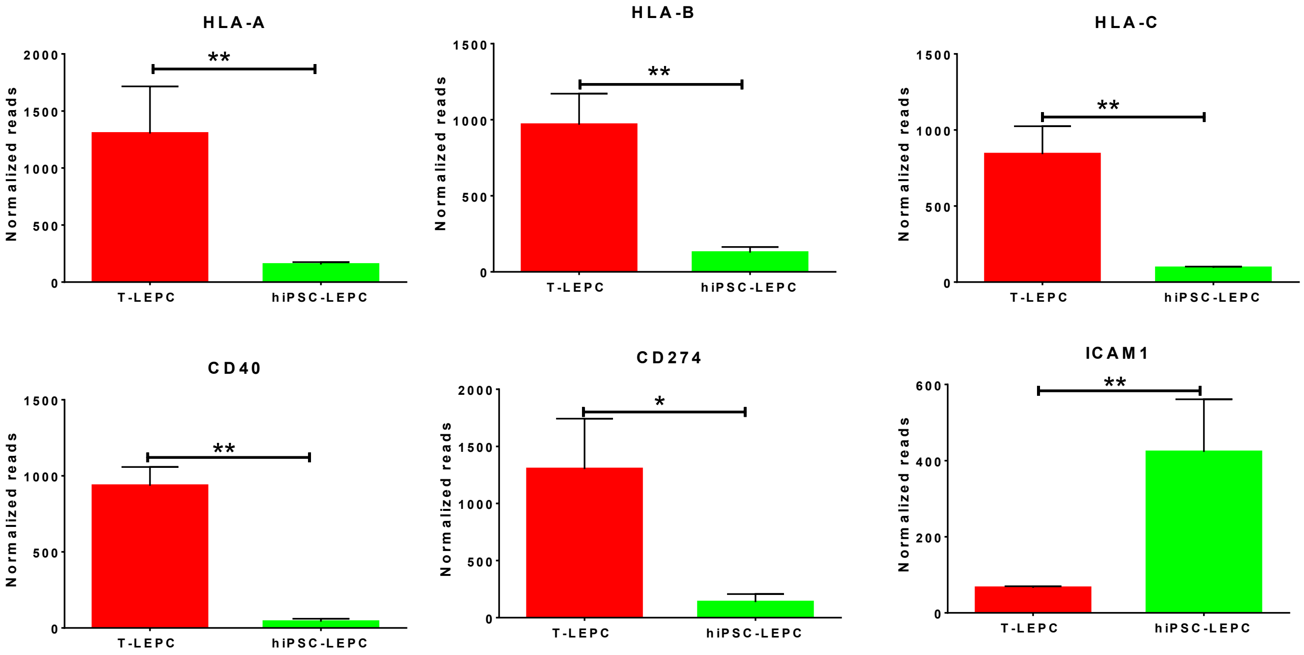

B

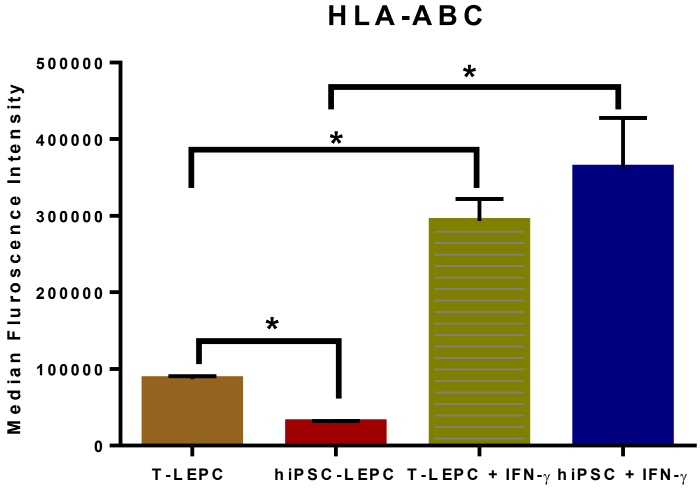

Figure S3: A) RNA sequencing data analysis of immune related genes expression in hiPSC-LEPC and T-LEPC. *p<0.05; ** p<0.01 B) Flow cytometric analysis shows median fluorescence intensity of HLA-ABC expression in T-LEPC and hiPSC-LEPC in presence or absence of Interferon-γ. Data are expressed as mean ± standard error of the mean of 4 individual experiments. *p<0.05.

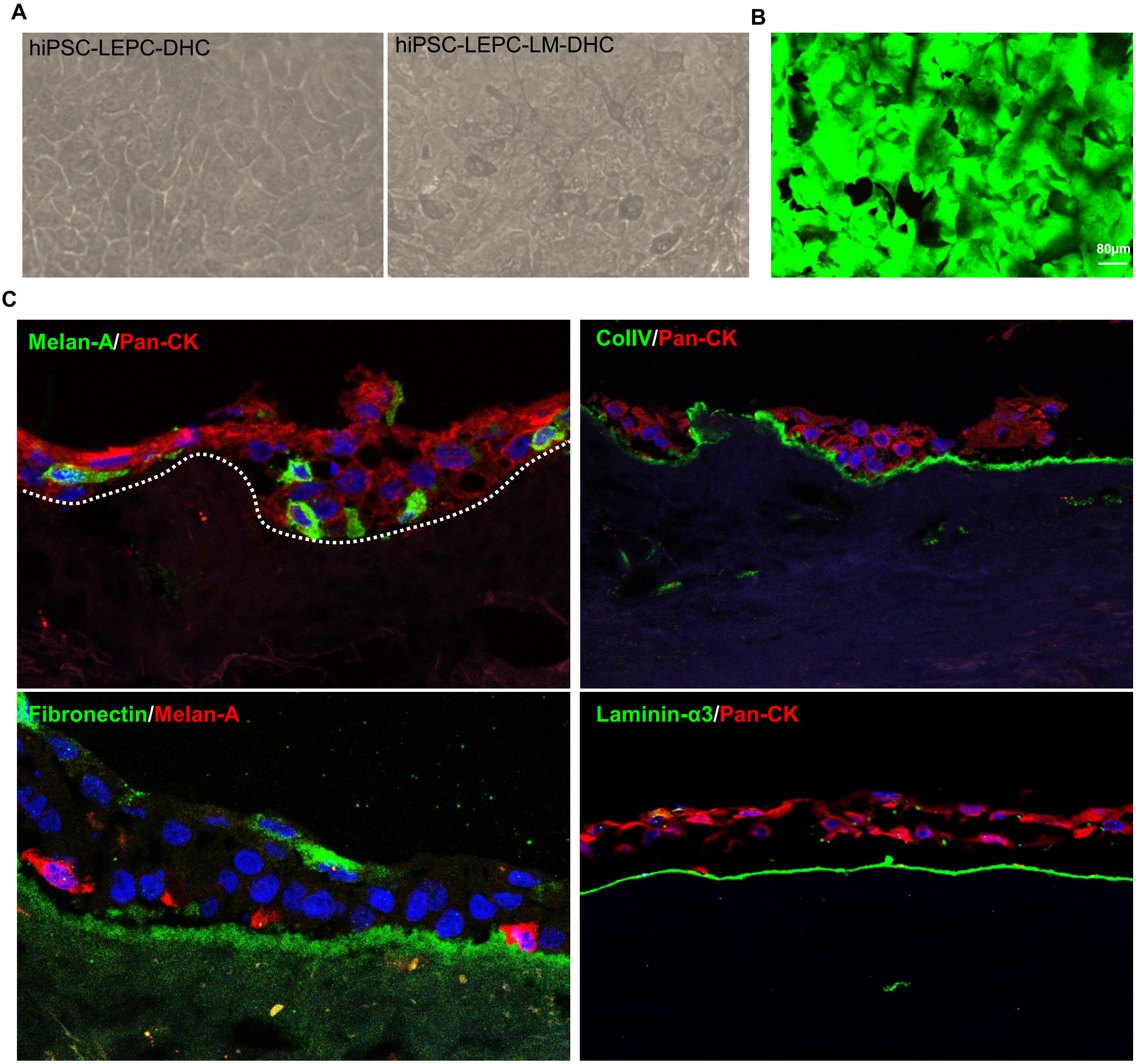

Figure S4. A: Phase contrast micrographs show the presence of hiPSC-LEPC and LM on the surface of decellularized corneal scaffolds. B) Live/dead staining of the hiPSC-LEPC/DHC scaffolds show the cells remained viable (green) after 48h of cultivation. **C)** Double immunofluorescence staining of DHC/L-hiPSC-LEPC-LM scaffolds showing the expression of cytokeratin (pan-CK, red) in all epithelial cells and Melan-A^{+} melanocytes (green) associated with basal cells (the dotted line represents basement membrane); intact fibronectin, col IV, and laminin-α3 expression (green)

S.no.	Age (Years)	Post mortem time (h)	Duration of cultivation (days)
1	75	12,08	24,52
2	60	42,17	27,44
3	68	15,14	26,34
4	68	15,14	29,84
5	54	30,25	31,43
6	78	39,93	28,54
7	71	67,42	18,70
8	71	67,62	24,50
9	66	26,57	18,50
10	66	26,57	21,50
11	74	23,87	24,78
12	65	38,50	23,18
13	78	60,63	21,43
14	78	60,63	22,43
15	74	32,17	21,52
16	68	28,92	18,53
17	59	25,16	16,56
18	79	24,19	33,46
19	87	28,75	25,47
20	87	26,38	31,14
21	92	45,26	18,04
22	81	37,34	34,86
23	81	36,72	32,55
24	75	23,64	35,46
25	59	12,24	31,37

Table S1: Organ cultured corneal scleral tissues used in this study

Antibody (clone), Host species	Antibody dilution	Application	Antibody source
Cadherin-E (24E10), Rabbit	1:200	Immunohistochemistry	Cell signaling
Cadherin-P (A-10), Mouse	1:100	Immunohistochemistry	Santa Cruz Biotechnology, INC.
Cadherin-P/Alexa488, Mouse	5 µl/10^6 cells	Flow cytometry	R&D systems
CD90 APC(5E10), Mouse	5 µl/10^6 cells<br>1:400	Flow cytometry<br>Immunohist/cytochemistry	BD Biosciences
CD117 PE (A3C6E2), Mouse	5 µl/10^6 cells	Flow cytometry	Miltenyi Biotec
CD117 PE (YB5.B8), Mouse	5 µl/10^6 cells	Flow cytometry	BD Pharmingen
CD200 Alexa-647 (OX-104), Mouse	5 µl/10^6 cells	Flow cytometry	Biolegend
Collagen Type IV (2F11), Mouse	1:200	Immunohistochemistry	SouthernBiotech
Cytokeratin pan (PCK-26), Mouse	1:500	Immunohistochemistry	Abcam
Cytokeratin 3/76 (AE5), Mouse	1:100	Immunohisto/cytochemistry	Millipore
Cytokeratin 12 (EPR17882), Rabbit	1:50	Immunohistochemistry	Abcam
Cytokerain 14 (LL002) Mouse	1:500	Immunohisto/cytochemistry	Abcam
Cytokeratin 15 (LHK15), Mouse	1:500	Immunohistochemistry	Abcam
Cytokeratin 15 (EPR1614Y), Rabbit	1:500	Immunohistochemistry	Abcam
Cytokeratin 17/19 (D4G2)	1:50	Immunocytochemistry	Cell Signaling
Fibronectin (IST-4), Mouse	1:100	Immunohistochemistry	Sigma-Aldrich
IgG2a, k, Isotype PE (MOPC-173), mouse	5 µl/10^6 cells	Flow cytometry	Biolegend
IgG3, k Isotype FITC (MG3-35), mouse	5 µl/10^6 cells	Flow cytometry	Biolegend
IgG2a, k Isotype APC (eBM2a), mouse	5 µl/10^6 cells	Flow cytometry	Invitrogen
ITGB4 PE (58XB4), Mouse	5 µl/10^6 cells	Flow cytometry	Biolegend
Ki-67 (EPR3610), Rabbit	1:500	Immunohistochemistry	Abcam
Laminin alpha 3 (546215), Mouse	1:200	Immunohistochemistry	R&D systems Biotechne
Melan A, (EPR20380), Rabbit	1:500	Immunohisto/cytochemistry	Abcam
DeltaN p63 (E6Q3O)	1:500	Immunohisto/cytochemistry	Cell Signaling
PAX6 (Poly19013), Rabbit	1:1000	Immunohistochemistry<br>Westernblotting	Biolegend
SSEA4 FITC (MC-813-70), Mouse	5 µl/10^6 cells	5 µl/10^6 cells	Biolegend
Vimentin, (D21H3), Rabbit	1:500	Immunohistochemistry	Cell Signaling

Table S2. List of antibodies used
